# Annotating the Insect Regulatory Genome

**DOI:** 10.3390/insects12070591

**Published:** 2021-06-29

**Authors:** Hasiba Asma, Marc S. Halfon

**Affiliations:** 1 Program in Genetics, Genomics, and Bioinformatics, University at Buffalo-State University of New York, Buffalo, NY 14203, USA; hasibaas@buffalo.edu; 2 Department of Biochemistry, University at Buffalo-State University of New York, Buffalo, NY 14203, USA; 3 Department of Biomedical Informatics, University at Buffalo-State University of New York, Buffalo, NY 14203, USA; 4 Department of Biological Sciences, University at Buffalo-State University of New York, Buffalo, NY 14203, USA; 5 NY State Center of Excellence in Bioinformatics & Life Sciences, Buffalo, NY 14203, USA

**Keywords:** arthropod, genomics, enhancer, cis-regulation, genome annotation

## Abstract

**Simple Summary:**

Insects comprise the largest and most diverse class of animals on earth, and have major impacts on human health and agriculture. The effort to better understand insect biology has led to the sequencing of hundreds of insect genomes. However, the usefulness of having a genome sequence is limited in the absence of a comprehensive annotation—a description of the function of each part of the sequence. Functional parts of the genome include not only genes, but also regulatory sequences that mediate gene expression. We discuss here methods used to identify regulatory sequences within the genome, with the emphasis on a pair of tools we have developed, REDfly and SCRMshaw, that can be used in tandem to carry out this task in an efficient and economical manner.

**Abstract:**

An ever-growing number of insect genomes is being sequenced across the evolutionary spectrum. Comprehensive annotation of not only genes but also regulatory regions is critical for reaping the full benefits of this sequencing. Driven by developments in sequencing technologies and in both empirical and computational discovery strategies, the past few decades have witnessed dramatic progress in our ability to identify *cis-*regulatory modules (CRMs), sequences such as enhancers that play a major role in regulating transcription. Nevertheless, providing a timely and comprehensive regulatory annotation of newly sequenced insect genomes is an ongoing challenge. We review here the methods being used to identify CRMs in both model and non-model insect species, and focus on two tools that we have developed, REDfly and SCRMshaw. These resources can be paired together in a powerful combination to facilitate insect regulatory annotation over a broad range of species, with an accuracy equal to or better than that of other state-of-the-art methods.

## 1. Introduction

Significant resources have been invested in sequencing insect genomes over the last decade, with over 768 species being fully or partially sequenced [1]. For instance, the i5k project was initiated to organize the sequencing and analysis of as many as 5000 arthropod genomes [2]. As valuable as these genome sequences are, however, sequencing a genome by itself is merely a beginning—the first step in a transformative process that builds on new information to generate fresh insights. Genomes are of limited value without comprehensive annotation: in addition to the DNA sequence itself, it is necessary to attach biological information to the genome, including not only the locations and identities of genes but also of non-coding regulatory elements. While gene annotation typically follows fairly quickly after assembly, regulatory annotation can be a long time in coming, if at all, and even then typically consists of putative locations only, without descriptions of function.

This lack of regulatory annotation is unfortunate, as regulatory sequences are the building blocks of transcriptional regulatory networks and essential for mediating both development and homeostasis [3,4]. Genes within a regulatory network need to be transcribed at the right time and in the right cells. This regulation is the result of the interaction of transcription factors (TFs) with specific *cis*-regulatory modules (CRMs, including but not limited to “enhancers”) that are frequently organized in a modular fashion to regulate specific spatiotemporal aspects of the expression of nearby genes. CRMs are typically a few hundred base pairs in length and are located upstream, downstream, or even within intronic regions of their target regulated gene, often at a considerable distance from the promoter.

One reason why regulatory annotation so often lags behind genome sequencing is that, historically, finding regulatory elements in the genome has been challenging even in well-studied model organisms because of their distant positions from target genes, the absence of a clear universal biochemical CRM marker, and the cell-type specificity of CRM activity [5,6,7,8]. For non-model organisms, where only limited functional genomic data tend to be available, regulatory annotation is even more difficult. In an effort to ameliorate this situation, we have developed two tools that facilitate CRM identification, in particular with respect to insect regulatory genomics. REDfly, the the Regulatory Element Database for *Drosophila* and other insects, is a comprehensive knowledge base of published insect CRMs. REDfly contains more than 25,000 experimentally validated *Drosophila melanogaster* CRMs associated with over 1700 genes, along with their sequences and the expression patterns for which they are responsible, accompanied by a growing number of CRMs identified in other insects [9]. SCRMshaw is a computational approach to Supervised *cis*-Regulatory Module discovery that can locate CRMs responsible for directing specific patterns of gene expression in a rapid fashion, with minimal required input [10,11,12]. Used jointly, these two tools have enabled us to identify CRMs across a range of insect species spanning over 345 million years of evolutionary divergence. In this paper, we briefly review current approaches to CRM discovery, and then show how REDfly and SCRMshaw together constitute a powerful platform for insect CRM discovery and regulatory genome annotation.

## 2. Empirical Approaches to CRM Discovery

### 2.1. Reporter Gene Assays

Traditionally, CRMs have been identified through empirical testing of genomic sequence fragments in reporter gene assays. This is a time-consuming and resource-intensive approach, as a CRM can sometimes reside hundreds of thousands of base pairs away from the gene that it regulates. Moreover, in vivo reporter gene assays are not well suited for genome-wide annotation, as they require the generation of a great many transgenic lines to be able to survey a sufficient length of the genome, many of which will provide negative results. More recently, with the availability of fully sequenced genomes and next-generation sequencing methods, high-throughput methods have been developed to functionally assay putative CRMs on a genomic scale (e.g., [13,14,15]). STARR-seq, which elegantly converts CRMs into their own reporters by cloning them downstream of a core promoter and sequencing the output, is one increasingly popular method [14,16,17,18,19,20]. Although reporter-based methods have long been viewed as a gold standard, due to the fact that they provide a direct functional readout of regulatory activity, there is growing recognition that these methods can lead to both false-positive and false-negative results [7,8,21]. However, the overall accuracy of reporter gene assays is believed to be high, and these remain the most definitive assays for regulatory function.

### 2.2. ChIP-Based Assays

CRM activity is strictly dependent on the binding of transcription factors, which makes it possible to use genome-wide methods that determine in vivo transcription factor binding sites for the prediction of active CRMs. One common method for genome-wide CRM discovery has been chromatin immunoprecipitation, followed by deep sequencing (ChIP–seq) [22,23]. ChIP-seq not only can be used to identify binding sites for specific TFs, but it can also be used to identify the in vivo binding sites of transcriptional coactivators present at large numbers of CRMs, such as the acetyltransferase p300. Coactivators do not bind to DNA directly but are recruited by TFs, and carry out various biochemical activities, ultimately leading to activation or repression. The advantage of this approach is that one does not need to know sets of relevant transcription factors a priori; the shortcoming is that focusing on a widely deployed coactivator does not allow for the preferential discovery of CRMs active in a specific tissue. Genome-wide chromatin profiling can also be obtained through ChIP-seq. For that purpose, antibodies against specific chromatin post-translational modifications are used. For example, histone H3, with its lysine at position 27 acetylated (H3K27Ac), shows a high correlation with active CRMs, whereas histone H3, with its K27 trimethylated (H3 K27me3), is indicative of inactive regions [24,25]. However, no single marker appears to universally distinguish CRMs.

Increased chromatin accessibility is also critical to facilitate precise gene regulation. Active CRMs reside in regions of open (nucleosome-depleted) chromatin, which can be identified on a genome-wide scale through a variety of methods. In particular, the formaldehyde-assisted identification of regulatory elements (FAIRE) [26,27,28], the assay for transposase-accessible chromatin using sequencing (ATAC-seq) [29], and DNase-seq [30] are all used to identify open chromatin regions across the genome. Improvements in single-cell technologies have led to the ability to measure chromatin accessibility using single-cell ATAC-seq (scATAC-seq), a potential game-changer in the ability to undertake cell-type-specific CRM discovery without needing to obtain large numbers of purified cells of uniform type [31,32,33,34,35,36,37].

### 2.3. Limitations

None of the abovementioned empirical methods are without limitations. They remain costly (compared to in silico approaches); can be difficult to validate, depending on the availability of biological resources such as cell lines, antibodies, and tissue samples, or the existence of relevant technologies, such as transgenesis; and carry false-positive and false-negative rates that can be surprisingly high—false-positive rates range as high as 40% for some ChIP-based methods [38,39,40] and from 10–20% for some ATAC-seq studies [31,32]. Moreover, CRMs may be functional only in certain cell types or under specific conditions, and thus can only be identified when assays include those cells or conditions. The features used by the abovementioned empirical approaches must, therefore, be assessed in multiple tissues over many developmental stages and/or under varying environmental conditions, in order to achieve comprehensive CRM discovery.

## 3. Computational Approaches

As part of the contemporary arsenal of methods for CRM discovery, computational approaches have proven to be an important complement to experimental ones. Computational CRM discovery has several potential benefits, including a low cost, rapid results, and no requirement for access to expensive and/or limiting biological resources and assays. This is of particular benefit when working with non-model organisms, for which there may be a genome sequence but frequently not extensive other resources. However, many current computational approaches still rely on experimental data for either training or as input (see below), which often negates these advantages.

### 3.1. Supervised Machine Learning

Recently, genome-wide computational CRM prediction has gained prominence with supervised machine-learning (ML) algorithms that are trained using one or more features from known CRMs. Features can consist of the DNA sequence itself, but frequently also include experimentally derived epigenetic information, such as histone post-translational modifications, transcription factor binding, and chromatin accessibility. In supervised ML, a classification algorithm is trained to distinguish between labeled positive and negative training examples (e.g., CRMs vs. non-CRMs) based on the features of these examples. The trained classifier is then used to predict the labels for uncharacterized input (e.g., unseen genomic regions). Among the ML approaches that have been used for CRM discovery are support vector machines (SVMs) [41,42], artificial neural networks (ANNs) [43], decision trees (DTs) [44], random forests (RFs) [45], probabilistic models (PGMs) [46,47], and, more recently, deep learning (e.g., [48,49,50,51,52]).

The commonly used SVM seeks to find a hyperplane in an N-dimensional space (where N equals the number of input features) that distinctly separates the data points. SVMs work very effectively when there is a clear margin of distinction between classes, but suffer when the data set is big, noisy, and/or overlapping, and they are prone to over-fitting.

Random forest classifiers, on the other hand, are an ensemble method that has become a popular machine-learning technique due to its ability to run efficiently on large datasets without over-fitting, and to deal with unbalanced and missing data [45]. Usefully, random forests can also indicate the relative importance of individual input features.

Deep learning algorithms are particularly suitable for dealing with large, high-throughput data sets. They utilize a hierarchical assembly of ANNs—nodes connected in a web acting like neurons in the brain, where connections between nodes are strengthened during training if they lead to successful outcomes—to carry out the process of machine learning. Due to their inherent non-linearity and high-level representation of features, algorithms using deep learning often outperform other ML-based methods in predicting CRMs.

### 3.2. Chromatin and Epigenetic Features

Examples of supervised learning tools based on chromatin features include chromatin signature identification by an artificial neural network (CSI-ANN) [43], ChromagenSVM [41], random forest-based enhancer identification using chromatin states (RFECS) [45], and deep learning for identifying *cis*-regulatory elements (DECRES) [49]. CSI-ANN uses an artificial neural network model to predict CRMs based on histone methylation and acetylation signatures, whereas ChromagenSVM employs a genetic algorithm to choose an optimum combination of histone epigenetic marks for use in an SVM-based classifier. Taking advantage of the strong feature-selection capabilities of random forests, Rajagopal et al. [45] evaluated the importance of different histone modifications using RFECS and an extended panel of histone-ChIP data, to conclude that a combination of three histone modifications, H3K4me1, H3K4me2, and H3K4me3, gave the strongest performance. However, almost as good a performance was achieved when substituting the more commonly assessed H3K27ac for H3K4me2. When all three of these aforementioned chromatin-based tools were applied to the same histone modification datasets in CD4^+^ T-cells, RFECS achieved the highest validation rate (70% vs. 57% for ChromaGenSVM and 51% for CSI-ANN) and lowest misclassification rate (7% vs. 27% and 35% as compared to ChromagenSVM and CSI-ANN, respectively). While in relative terms RFECS, therefore, gives the strongest performance, the actual validation rates should be viewed with caution, as the study used an extremely generous criterion for true positive predictions, defined as falling with ±2.5 kb of a set of themselves non-dispositive CRM markers, including DNaseI hypersensitive sites and the binding of several TFs and coactivators [49]. The deep-learning approach, DECRES, uses a comprehensive feature set integrating histone modification, TF binding, DNase-seq, FAIRE-seq, and ChIA-PET data from the ENCODE project [53], along with the transcriptionally active enhancers and promoters cataloged by the FANTOM project [54], to predict CRMs. When evaluated against two empirically determined CRM datasets from K562 cells, DECRES displayed high sensitivity (predicting 65% and 98% of validated CRMs from the two sets, respectively) but also a high false-positive rate (predicting 53% and 92% of non-CRMs) [49].

### 3.3. Sequence Features

Although substantial amounts of cell-type-specific epigenetic data exist for human and mouse such data are often much more limited for other organisms, and reliance on extensive experimental data nullifies many of the advantages of having a computational approach in the first place. Therefore, a number of methods have explored using sequence features alone as a basis for CRM prediction. Common features used in sequence-based approaches are DNA subsequence composition (e.g., kmers) or TF binding site motifs, although additional features such as G + C and A + T frequencies or CpG island length have been used as well [42]. Kmer-based methods have often been coupled with SVM classifiers, e.g., “kmer-support vector machine” (kmer-SVM) [55]. Kmer-svm can accurately predict CRMs (defined by the binding of the p300 co-activator) from genomic sequence alone and can also discriminate CRMs from non-functional elements with high accuracy in a cross-validation framework. This approach was subsequently improved by “gapped kmer SVM” (gkm-SVM), which takes kmers with gaps into consideration [56].

Deep-learning approaches have been increasing in popularity as the underlying methods become more mature and as advances in computing power make them more feasible. DECRES (introduced above) can be trained and applied using only sequence features, although its performance is then significantly reduced, compared to training with a full range of experimental chromatin-level data [49]. BiRen [52] leverages the deep learning power of convolutional neural networks (CNNs) and bidirectional recurrent neural networks (BRNNs) to predict CRMs using DNA sequences alone as input, although chromatin and histone modification data are used during training. Although this approach exhibited high performance in a cross-validation setting, validation based on overlap with genomic features suggestive of CRMs was relatively weak: association with DNaseI hypersensitive sites, histone H3K27ac, and high-occupancy target for TF ChIP (HOT) regions was only 55%, 40% and 23%, respectively. Nevertheless, BiRen outperformed competing methods also based solely on sequence-feature input [42,55] in head-to-head comparisons, showing the promise of contemporary deep-learning approaches for CRM discovery.

Unsupervised machine-learning methods have suggested the presence of different CRM classes, such as “strong” and “weak” enhancers, based on histone modifications and other ChIP-derived data [46,47]. Although reporter-gene-based validation has called these specific designations into question [13], the sequences themselves are independently discoverable using supervised methods based solely on sequence features. iEnhancer-EL [57] uses a combination of sequence features to build a two-layer ensemble model formed from 16 individual SVM classifiers. In testing, it was shown to perform well at both CRM detection and at stratifying the identified CRMs by their ChromHMM-defined [46] “weak” and “strong” designations, with a measured accuracy of ~75% for the CRM discovery task. This was modestly improved upon (77% accuracy on the same data set) by Nguyen et al. [58], whose iEnhancer-ECNN replaces the ensemble SVM model with an ensemble CNN model instead, using a combination of one-hot encoding and kmers as the sequence input. Another recently proposed method, iEnhancer-5Step, makes use of a word-embedding approach borrowed from natural language programming along with an SVM classifier [59]. This model showed further improvements—an accuracy of 79%—on the same data set analyzed by the other “iEnhancer” methods. However, it is important to note that all of these methods have only been tested using a pre-classified set, with equal numbers of positive and negative sequences (as defined by ChromHMM). This raises significant questions as to what their performance would look like in a “real-world” test using a complete genome, where negative sequences significantly outnumber positive ones, and if validated against a set of CRMs defined using orthogonal criteria.

## 4. Cross-Species or Non-Model Insect CRM Discovery

CRM discovery in non-traditional insect models presents a particular challenge. Researchers studying these insects often lack the backing of large well-established communities, able to dedicate “big science” resources to gathering the necessary extensive genomic data, and many methods, in particular transgenesis, are not well-established outside of the primary research organisms. Recent advances in technologies that appear to be broadly applicable, such as CRISPR-Cas9 genome manipulation, as well as rapidly declining costs for accessing sequencing-based approaches such as ATAC-seq, are helping to level the playing field [29,60]. Single-cell methods, especially scATAC-seq, show great promise as they should help to get around the problem of needing to purify large numbers of individual cell types in order to obtain chromatin profiles [31,32].

Computational methods that can be rapidly and inexpensively applied are, in principle, an attractive solution for obtaining a quick regulatory annotation of newly sequenced insect genomes. However, as demonstrated in the preceding discussion, most effective computational methods still require input data, either in the form of training data using known CRMs, or chromatin-level genomic assays. Paradoxically, therefore, computational CRM discovery appears to be dependent on just the sorts of empirical studies that it is intended to bypass.

A potential way past this dilemma is to leverage data from one species to train a computational algorithm to search the genome of a second species [48,61,62,63]. Such an approach has two basic requirements. First, there must be sufficient similarity in sequence-level CRM properties among species for a cross-species approach to be feasible, as the only input data for the second species may be its genome sequence. This requirement raises a biological question: even when there is a lack of obvious sequence similarity, do functionally related CRMs contain shared features? A growing body of literature suggests that this is the case, at least for CRMs regulating genes involved in core developmental processes. For instance, the similarity of the co-occurrence of sequence patterns has been used to make use of known *Drosophila melanogaster* CRMs to identify “orthologous” CRMs in the distantly related drosophilid and sepsid fly species [61]. The Capra lab has used two machine-learning frameworks (SVMs and CNNs) to distinguish CRMs from the genomic background, based on DNA sequence patterns, and models trained to predict CRMs from one species also accurately identify CRMs in the same cellular context in other species—from humans to opossums [48]. While these sets of species maintain a reasonable amount of alignable sequence, cross-species CRM prediction has also been demonstrated across more highly sequence-diverged species pairs. Minnoye et al. [63] designed a multi-class neural network-based method, DeepMEL, that when trained on human melanoma ATAC-seq data successfully predicted enhancers for two related but distinct cell types across six different species (human, dog, horse, pig, mouse, zebrafish); the latter pairing begins to approach the level of divergence observed in family-level comparisons among the holometabola [64]. Transcription factor binding site clustering, based on *Drosophila melanogaster* CRMs, has been used to discover CRMs in other holometabolous insects [65,66,67,68], and the SCRMshaw algorithm (described more fully below) has used *Drosophila* data to successfully identify CRMs in species as distantly diverged as the Hemiptera e.g., [62,69,70] (H.A. and M.S.H., unpublished data), based on statistical similarities in subsequence (kmer) counts among the CRMs. Detailed analysis of such distant but related CRMs has revealed the presence of common sets of transcription factor binding sites, and even potential enhancer grammars (conserved arrangements of binding sites), shared features that blend into the noise of whole-genome analyses but that become possible to detect once CRM locations have been defined [62]. So far, most success in cross-species CRM discovery where genomes are not alignable has been seen with CRMs that function in well-conserved developmental pathways, and it remains to be determined how widespread these deep sequence-level homologies are with respect to less fundamental regulatory networks.

The second requirement for cross-species CRM discovery is that there must be an extensive enough body of data of a suitable type in the training species. Sets of known CRMs are ideal for this purpose, as this is purely sequence-based data that can be readily applied to other sequenced genomes. Here insects have a significant advantage, due to the extensive set of known CRMs available for *Drosophila melanogaster*, many of which are functionally validated. Moreover, the majority of these have been carefully curated into the insect-specific REDfly database, a comprehensive source of regulatory data unique among the metazoa [9]. In the remaining sections of the paper, we focus on how REDfly and SCRMshaw can be used together for CRM discovery across multiple insect species as a powerful platform to facilitate the field of insect regulatory genomics.

## 5. REDfly and SCRMshaw: Powerful Tools for Insect Regulatory Genomics

### 5.1. REDfly

REDfly is a one-stop curated knowledgebase for insect *cis*-regulatory data [9]. Historically focused on experimentally verified *Drosophila melanogaster* CRMs, REDfly has grown over its 15 years of existence to include *Drosophila* transcription factor binding sites, CRMs identified from epigenetic profiles and computational prediction, and, most recently, CRMs from an increasing number of other insects, including important disease vectors such as mosquitoes. (Currently, three insect species in addition to *D. melanogaster* have been incorporated—*Anopheles gambiae, Aedes aegypti*, and *Tribolium castaneum*—with more on the way.) To date, REDfly has over 25,500 *D. melanogaster* CRMs (60% from in vivo reporter genes, most of the remainder from cell-culture assays) associated with 12–15% of protein-coding genes, and ~2700 defined transcription factor binding sites (TFBSs). These data are based on more than 1200 curated publications. The core REDfly *Drosophila* CRM annotations are provided to FlyBase, making *Drosophila* the only model organism whose genome annotation provides comprehensive coverage of validated CRMs, enabling direct integration with other *Drosophila* genomic and genetic data.

**Figure 1:**
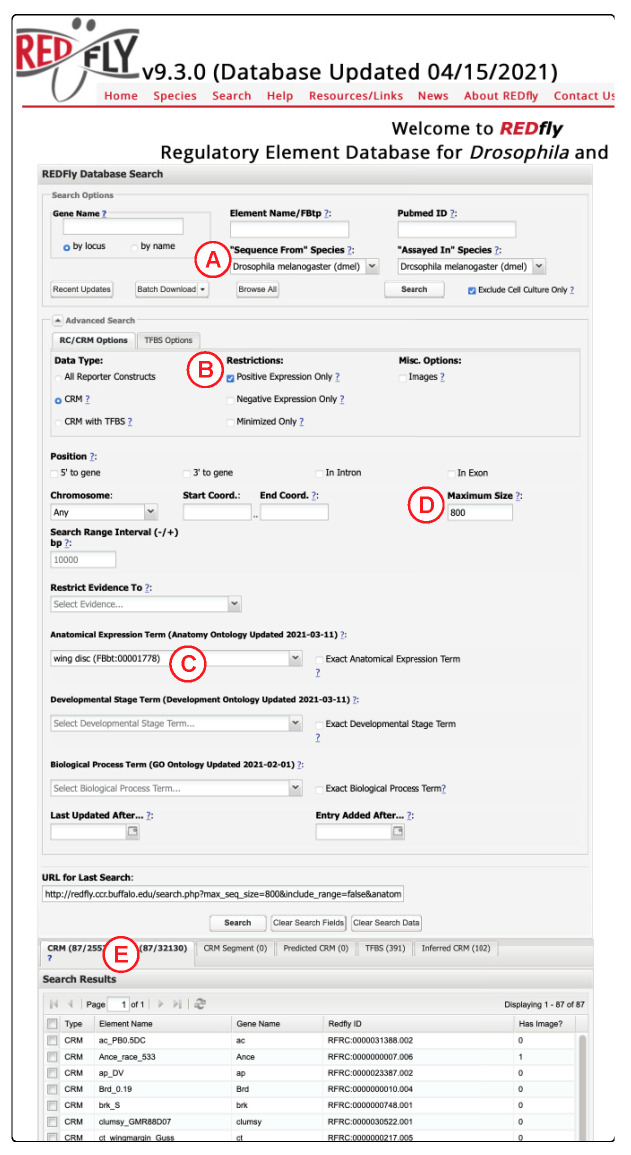
The REDfly search interface. Researchers can make use of REDfly’s comprehensive search capabilities to assemble sets of CRMs with specific properties. In this example, for instance, over 25,000 “CRM” records will be searched for (**A**) sequences belonging to the *Drosophila melanogaster* genome that (**B**) positively regulate gene expression in the (**C**) wing imaginal disc, and that are (**D**) no greater than 800 bp in length. Results are listed in the Results Table (**E**). Clicking on an individual result opens a Detailed Results window with extensive further information, or multiple records can be selected using the checkboxes for download in a variety of formats.

One strength of REDfly is the extensive detail it provides about the CRMs it curates (Figure 1). Regulatory activity is described using the *Drosophila* anatomy and development ontologies [71], which allows for retrieval of tissue-specific and stage-specific CRM datasets at multiple degrees of granularity. Terms from the Gene Ontology [72,73] are incorporated to annotate regulatory elements that respond to specific physiological or environmental cues (e.g., wound-healing, hypoxia). CRMs can be filtered by size, genomic location, position relative to target genes (e.g., upstream, downstream, intronic), and sex-specificity. Overlapping regions between multiple CRMs are automatically calculated to suggest potential minimal CRM sequences and their regulatory activity.

As the most detailed existing platform offering regulatory element annotation for any animal, REDfly serves as an important platform for supporting both empirical and computational research. REDfly has contributed to numerous studies in multiple areas relating to non-*Drosophila*, as well as *Drosophila* systems including studies of basic CRM biology (e.g., [74,75,76,77,78,79,80,81,82,83]) and interpretation of genomic data such as TF binding studies (e.g., [84,85,86]), studies of insulators [87,88], of chromosome domains and “states” (e.g., [89,90,91,92]), of 3D-chromatin conformation [93,94], and of ncRNA and eRNA expression [95,96]. REDfly data have facilitated the validation of ATAC-seq approaches (e.g., [97,98]), have been used to establish TF-CRM associations for the study of gene regulatory networks [74,99,100,101,102,103,104], and have enabled studies of CRM evolution and TFBS turnover (e.g., [104,105,106,107,108,109,110,111]).

REDfly has also played a dramatic role in developing methods for computational CRM discovery. Its extensive collection of experimentally verified CRMs provides a ready source of validation data for assessing CRM predictions and for comparing among methods [21,112,113,114,115,116]. Perhaps more importantly, REDfly’s advanced search and filtering features make it an unmatched source for compiling training data for machine-learning approaches [10,11,61,117]. As we review in the following section, we have used REDfly to develop nearly 50 individual training sets, spanning numerous *Drosophila* tissues and time points, for use in conjunction with our SCRMshaw algorithm. This has enabled considerable new CRM discovery in *Drosophila* but, more excitingly, also allows for cross-species identification of CRMs in a wide range of insect species.

### 5.2. SCRMshaw

The CRM training data made available by REDfly enabled us, in collaboration with Saurabh Sinha’s laboratory at the University of Illinois, to develop SCRMshaw (for supervised *cis*-regulatory module discovery), a highly effective method for computational CRM discovery [10,11,12]. Other than the possession of a moderately sized training set of known CRMs—we have had success with fewer than ten CRMs, although 20–30 is preferred—the only input SCRMshaw requires is an annotated genome. As no other genomic data are required (e.g., no binding site data, chromatin conformation or state data, or histone modification data), SCRMshaw is ideal for use with newly sequenced and less-studied insect species.

**Figure 2:**
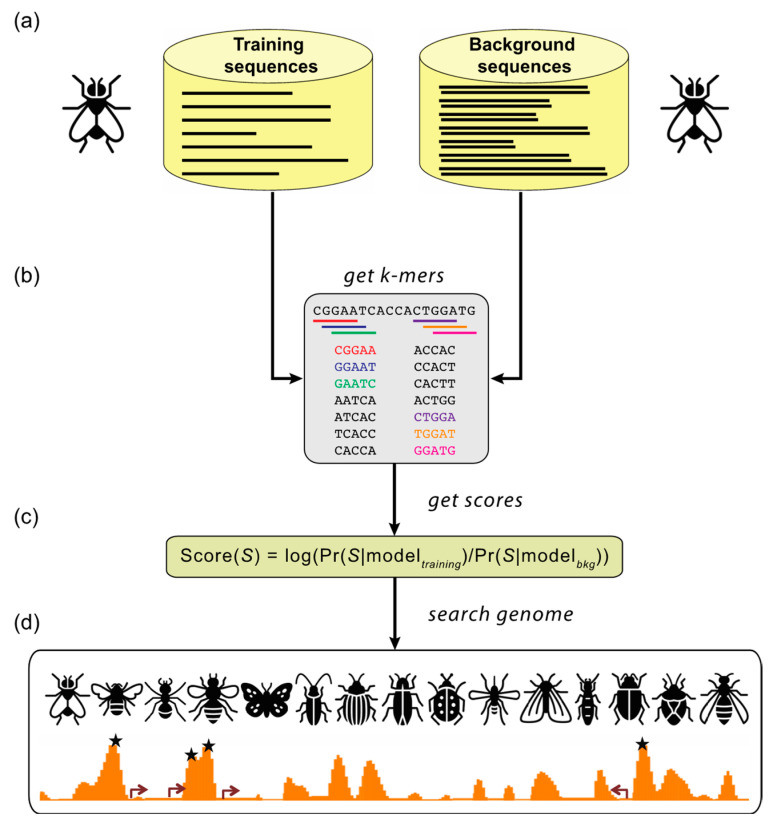
Supervised motif-blind CRM discovery (SCRMshaw). (**a**) SCRMshaw uses a training set of known *Drosophila melanogaster* CRMs (“training sequences”), drawn from REDfly, that are defined by common functional characterization, and a 10-fold larger background set of similarly sized non-CRM sequences (“background sequences”). (**b**) The short DNA subsequence (*kmer*) count distributions of these sequences are then used to train a statistical model. The trained model (**c**) is used to score overlapping windows in the “target genome”; to date, we have successfully used multiple different species from the Holometabola and several Hemiptera. (**d**) High-scoring regions are predicted to be functional regulatory sequences (asterisks). Figure adapted from [69]. Insect images downloaded from TheNounProject.com (accessed on 28 April 2021) set “Bugs” by Georgiana Ionescu, under the CC-BY license.

SCRMshaw (Figure 2) uses a training set composed of known CRMs, defined by a common functional characterization (e.g., “nervous system”, “midgut”) to build a statistical model that captures their short DNA subsequence (kmer) count distribution. This kmer distribution is then compared to that of a set of non-CRM “background” sequences in a machine-learning framework. The kmers likely serve as proxies for the unknown TFBSs, but TFBSs themselves, even when known, are not explicitly used by the algorithm. The trained model is then used to score overlapping sequence windows in the genome, and the highest-scoring windows are output as predicted CRMs. SCRMshaw has proven to be remarkably effective: when SCRMshaw predictions are tested empirically using reporter gene assays, success rates have averaged ~80%, with some training sets yielding over 90% true positives [10,11,62,69,118].

### 5.3. Cross-Species Prediction

As noted above, SCRMshaw’s true power is its ability to predict CRMs across species. Kazemian et al. [62] used SCRMshaw with *Drosophila* CRM training data to discover CRMs across the entire ~345 Mya range of the holometabolous insects. By using the same methods and training sets as previously used for within-species CRM discovery in *D. melanogaster* [10], and instead searching the genomes of *Anopheles gambiae, Aedes aegypti, Tribolium castaneum, Apis mellifera, and Nasonia vitripennis,* SCRMshaw successfully predicted CRMs in a cross-species fashion with an approximately 75% prediction success rate, based on reporter gene assays in xenotransgenic flies [62,69,118]. Direct testing of a predicted *Tribolium* CRM in transgenic *Tribolium* confirmed that SCRMshaw can find bona fide CRMs cross-species [70]. Preliminary data using hemipteran genomes suggest at least some ability to predict CRMs in this even more diverged insect order as well (H.A. and M.S.H., unpublished data).

### 5.4. Training Set Improvement

As with any machine-learning method, a key factor affecting SCRMshaw’s importance is the quality of the training data. Interestingly, our testing has shown that even training sets made up of randomly grouped but validated CRMs outperform groups of random non-coding, non-regulatory sequences when used for SCRMshaw training [10,21]. This is most likely due to the increased presence of binding sequences for common transcription factor families (e.g., E-boxes, homeodomain binding sites) in the true CRM sequences, and may account for the positive predictive performance obtained even with our least effective training sets. However, increasing the cohesiveness of the training sets, so that they fully represent groups of CRMs with common activity profiles, should improve predictive performance.

We have constructed over 48 training sets spanning a broad range of gene expression patterns across tissues and stages, using the most current available CRM data in the REDfly database. For the most part, these new sets are generated automatically by filtering REDfly CRMs based on anatomical classifications derived directly from the *Drosophila* anatomy ontology. Because the anatomy terms are not all temporally specific—while some terms distinguish between embryonic, larval, and adult stages, others do not—some of the training sets are likely too broadly constituted, leading to reduced performance. Taking advantage of updated temporal staging data in REDfly [9], as well as performing manual refinement to create more specific groupings of known CRM-driven expression patterns, should help to improve training set quality. We recently developed a training set evaluation pipeline, *pCRMeval*, that enables unbiased assessments of SCRMshaw performance on a training set by training set basis, to aid in this process [21].

**Figure 3:**
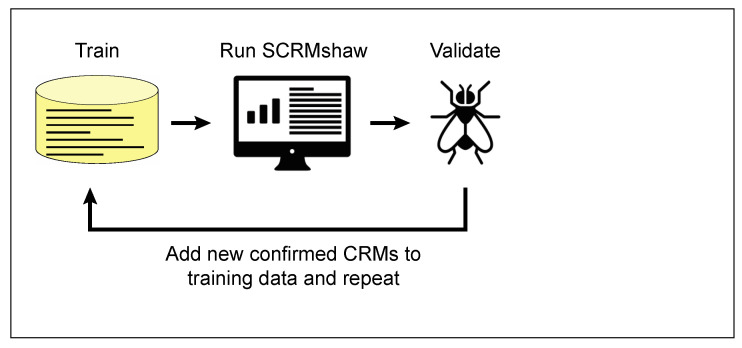
Iterative search and validation. Sequences validating as true CRMs from an initial SCRMshaw search can be added back to the training set, and the new, enlarged training set used for a subsequent round of predictions. This strategy can effectively compensate for an initially weak training set. Images downloaded from TheNounProject.com (accessed on 28 April 2021), “Fly” by Georgiana Ionescu, and “analytics” by Wilson Joseph, under the CC-BY license.

What can be done when a cohesive enough set of training CRMs of sufficient number is not available? In such cases, an iterative search-and-validation strategy has proven very effective (Figure 3). For example, in collaboration with Thomas Williams’ laboratory at the University of Dayton, we used SCRMshaw on a small training set of just seven CRMs that drive gene expression in the *Drosophila* adult abdomen, in a first round of SCRMshaw, to identify CRMs potentially involved in abdominal pigmentation. Empirical testing of 18 of these predictions revealed 10 new CRMs regulating the desired specific expression pattern (55%); an additional three appeared to be bona fide CRMs but with a different expression profile (for a total of 13/18 or 72% validating as CRMs). The ten CRMs driving the “correct” expression pattern were then combined with the original seven members of the training set to generate a new, 2.5-fold expanded training set for a second round of SCRMshaw prediction. Notably, the top prediction results from this second round did not include seven of the eight sequences previously found to be false positives by empirical testing—including true CRMs with non-targeted expression profiles—but still contained all ten of the previous true positives. Empirical validation for the second round of predictions confirms this improved performance: out of 21 CRMs tested, 20 were functional CRMs (95%) of which 17 (81%) had the expected pattern of expression (T. Williams, personnal communication). These results demonstrate that iterative approaches can serve to augment weak training sets to improve true-positive: false-positive ratios and underscore the importance of having a well-constructed training set. Although so far we have used this approach exclusively for *Drosophila* CRM prediction, iterative searching should also be useful for increasing the success of our cross-species predictions. Validated cross-species sequences can be added to our training sets in the same way that we have added validated *Drosophila* CRMs, with similarly improved results. Indeed, we expect that we may see even more dramatic improvement in some cases, as by adding in additional sequences from the species being searched, we will be increasing the number of same-species sequences, moving the SCRMshaw search closer to a same-species search.

### 5.5. Limitations

While many insect species are being sequenced, the genome assemblies and annotations are of varying quality, ranging from extremely well-assembled genomes (e.g., *Aedes aegypti* with a scaffold N50 size of 409,777,670 bp) to very poorly assembled genomes (Yellow Sally stonefly with a scaffold N50 of only 457 bp) [119,120]. How do these factors affect CRM prediction using SCRMshaw? Since, by default, SCRMshaw searches the genome in 500 bp windows, we reasoned that assembly is not likely to be a major limiting factor for any but the most poorly assembled genomes. By simulating different degrees of genome assembly for the *Drosophila* genome, and comparing the results to those obtained with the fully assembled genome, we determined that this is indeed the case: SCRMshaw maintains its strong predictive power when applied to increasingly less well-assembled genomes, with only a minor drop-off in sensitivity—less than 15% on average—and a negligible increase in the false-positive rate [21]. Assembly quality does not, therefore, appear to present a significant barrier to successful CRM prediction using SCRMshaw. Moreover, with long-read sequencing technologies becoming more prevalent, less fractured assemblies are now frequently available [121].

The second factor to consider is genome annotation, which can lag considerably behind sequencing and assembly: only 40% of all i5k-sequenced species currently have an accompanying predicted gene set [120]. Because SCRMshaw looks at kmer-level patterns, we typically exclude coding sequences from the analysis out of concern that the inherent constraints on coding sequences (e.g., codon biases and the limited number of valid codon triplets) will affect the SCRMshaw scoring. To test how important this is, we have compared the results of running SCRMshaw on the *Drosophila* genome with and without exons masked (H.A. and B. Yuen, unpublished data). These tests show that there is only minimal overlap in the top predictions between the masked and unmasked versions, confirming the importance of excluding coding sequences from the analysis. Therefore, having a good draft gene annotation is an important requirement for ensuring optimal SCRMshaw predictions. Fortunately, common genome annotation pipelines such as Maker2 [122] and Braker2 [123] are generally effective at detecting protein-coding regions, enabling adequate initial annotations to be generated.

## 6. Integrating Computational and Experimental Approaches

With empirical methods such as ATAC-seq becoming increasingly more accessible and affordable, it is fair to ask the question, what is the future of computational CRM prediction for insect genomes? We believe that the combination of REDfly and SCRMshaw retains several powerful advantages. For one, the approach remains both rapid and cost-effective. With no required access to biological samples, negligible expense, and only a few days’ time—much of which is hands-off—a reasonable first-pass annotation of the regulatory genome is possible for any recently sequenced insects within the holometabola, and probably their nearest neighboring families. This may prove especially useful for acquiring a broad sampling of CRM data for evolutionary studies; because SCRMshaw uses the same training data to search each species, the chances of discovering homologous (or functionally similar) CRMs for orthologous genes are high. In the long run, however, the greatest benefit is likely to be seen from combining computational prediction with other forms of genomic CRM discovery, where the in silico results can help to sharpen and refine empirical data. For instance, computational CRM discovery with subsequent alignment across groups of moderately closely related species can pinpoint important sequence motifs, and provide insights into possible enhancer grammar [62]. When used in conjunction with open-chromatin assays, SCRMshaw can help distinguish CRMs from non-CRM open regions, and, for whole-animal assays, is able to help home in on the most relevant tissue-specific CRMs. When used with single-cell methods, the fact that SCRMshaw predictions are based on tissue-specific training sets will help to assign identities to individual cell types to better interpret the single-cell results, and may help to develop improved methods for distinguishing sets of CRMs that are most closely related (i.e., integrate similar transcription factor inputs) from those that use different input strategies to achieve the same regulatory output (e.g., [124,125]). Chromatin profiling and next-generation sequencing assays each contain various biases (e.g., [126,127,128,129]). Those that are known can be corrected for to a certain extent, whereas other biases may not yet be well understood. Combining such assays with SCRMshaw, a wholly orthogonal method, should help in weeding out false-positive results from both types of assays, leading to more accurate CRM prediction overall.

## 7. Conclusions

The rapid growth in sequenced insect genomes requires the development of equally rapid and economical means for annotating the regulatory components of these genomes. Although many methods for CRM identification rely on extensive empirical data, a subset of computational approaches, including SCRMshaw, function effectively using sequence features only as input for both training and discovery. We discussed here how SCRMshaw, using the wealth of *Drosophila* regulatory data curated by REDfly, can be used cross-species to produce reasonable first-draft annotations of regulatory sequences throughout the holometabola and likely beyond. We focused on SCRMshaw because that is where data demonstrating good cross-species performance currently exist; however, it is likely that other sequence-based CRM discovery methods will similarly be capable of cross-species discovery. Our experience suggests that the most critical factor is the quality and cohesiveness of the training data. As the CRM data in REDfly become more finely annotated for developmental timing and cellular identities, it should be possible to generate improved training data and subsequently, an ever more accurate prediction of CRM locations in newly sequenced genomes. Moreover, as data for CRMs accumulate in more phylogenetically basal species, the ability to push prediction success into further diverged insect orders may become feasible.

Computational pipelines are able to provide rapid first-pass gene annotations of newly sequenced genomes, but require experimental follow-up in order to refine gene models for a final, accurate, finished annotation [130]. In the same way, REDfly and SCRMshaw can be paired as a powerful combination to generate initial, albeit imperfect, regulatory annotations for insect genomes, to be further refined by subsequent empirical CRM identification.

## 8. URLs

*REDfly* is freely accessible to the public at http://redfly.ccr.buffalo.edu.

*SCRMshaw* software and associated useful utility programs can be downloaded from the Halfon lab GitHub site at https://github.com/HalfonLab. Protocols for using SCRMshaw can be found in references [12] and [21].

## Abbreviations

ANN	Artificial neural network
ATAC-seq	Assay for transposase-accessible chromatin using sequencing
auROC	Area under the receiver operating characteristic
BRNN	Bidirectional recurrent neural networks
ChIA-PET	Chromatin interaction analysis with paired-end tag
ChIP	Chromatin immuno precipitation
CNN	Convolutional neural network
CRM	*cis*-regulatory module
CSI-ANN	Chromatin signature identification by artificial neural network
DECRES	Deep learning for identifying *cis*-regulatory elements
ECNN	Ensemble of CNN
ENCODE	Encyclopedia of DNA elements
FAIRE	Formaldehyde-assisted isolation of regulatory elements
FANTOM	Functional annotation of the mammalian genome
Gkm-SVM	Gapped kmer support vector machine
HMM	Hidden Markov model
Kmer-SVM	kmer-support vector machine
ML	Machine learning
REDfly	Regulatory element database for *Drosophila*
RF	Random forest
RFECS	Random forest-based enhancer identification using chromatin states
ROC	Receiver operating characteristic
scATAC-seq	Single cell assay for transposase-accessible chromatin using sequencing
SCRMshaw	Supervised *cis*-regulatory module discovery
STARR-seq	Self-transcribing active regulatory region sequencing
SVM	Support vector machine
TF	Transcription factor
TFBS	Transcription factor binding site
